# The 14q32 maternally imprinted locus is a major source of longitudinally stable circulating microRNAs as measured by small RNA sequencing

**DOI:** 10.1038/s41598-019-51948-6

**Published:** 2019-10-31

**Authors:** Gabriel N. Valbuena, Sophia Apostolidou, Rhiannon Roberts, Julie Barnes, Wendy Alderton, Lauren Harper, Ian Jacobs, Usha Menon, Hector C. Keun

**Affiliations:** 1 Division of Cancer, Department of Surgery and Cancer, Imperial College London, London, W12 0NN United Kingdom; 2 MRC Clinical Trials Unit at UCL, Institute of Clinical Trials & Methodology, University College London, Gower Street, London, UK; 3 Abcodia Ltd, PO Box 268, Royston, SG8 1EL Hertfordshire UK; 4 Early Detection Programme, Cancer Research UK Cambridge Centre, University of Cambridge, Cambridge, UK; 5 Cancer Research UK, Angel Building, 407 St John Street, London, UK; 6 University of New South Wales, Sydney, New South Wales Australia

**Keywords:** miRNAs, Biomarkers

## Abstract

Understanding the normal temporal variation of serum molecules is a critical factor for identifying useful candidate biomarkers for the diagnosis and prognosis of chronic disease. Using small RNA sequencing in a longitudinal study of 66 women with no history of cancer, we determined the distribution and dynamics (via intraclass correlation coefficients, ICCs) of the miRNA profile over 3 time points sampled across 2–5 years in the course of the screening trial, UKCTOCS. We were able to define a subset of longitudinally stable miRNAs (ICC >0.75) that were individually discriminating of women who had no cancer over the study period. These miRNAs were dominated by those originating from the *C14MC* cluster that is subject to maternal imprinting. This assessment was not significantly affected by common confounders such as age, BMI or time to centrifugation nor alternative methods to data normalisation. Our analysis provides important benchmark data supporting the development of miRNA biomarkers for the impact of life-course exposure as well as diagnosis and prognostication of chronic disease.

## Introduction

MicroRNAs (miRNAs) are a class of highly conserved, small non-coding RNAs (typically around 22nt long) that play a role in post-transcriptional regulation of gene expression^1^. They guide RNA-induced silencing complexes (RISCs) to the 3′-untranslated region (UTR) of mRNAs with complementary sequences, leading to the downregulation of gene expression either through translational repression or mRNA degradation^2^. miRNAs are ubiquitously expressed across different cell types, and help control a wide range of physiological processes, including cell-cycle control, cell differentiation, metabolism, and apoptosis^3–6^. Distinctive signatures of microRNA dysfunction have also been observed in a range of conditions, including neurodegenerative disease^7^, cardiovascular disease^8^, and cancer^9,10^.

The characteristic microRNA profiles associated with specific pathologies has led to extensive investigation into microRNA biomarkers of disease. Significant amounts of miRNA have been found in extracellular human biofluids including blood plasma^11^, and are remarkably stable despite the presence of endogenous RNAses in circulation. This presents opportunities to develop non-invasive diagnostic or prognostic tests, as biofluids are easier to sample than tissue. Circulating miRNA biomarkers have been identified for conditions such as various cancers^11,12^, brain injury^13^, and diabetes^14^, and the literature on the matter continues to expand rapidly.

Most published work on circulating miRNA biomarkers use a case-control study design, often with a single timepoint, small sample sizes and varying levels of case-control matching^15^. These limitations contribute to the poor track record of reported biomarkers entering clinical practice, as few achieve the anticipated performance in follow-up studies^16–18^. While there are notable exceptions that have been successfully validated, such as the use of circulating miR-371a-3p and other members of the miR-371–373 cluster in germ-cell tumors^19,20^, there are currently still no circulating microRNA-based diagnostic or prognostic tests approved for use by the U.S. Food and Drug Administration. A fundamental cause of this failure to translate is insufficient consideration of variability in the healthy population. Underestimating the extent of natural variation can lead to the selection of candidate biomarkers that are either insufficiently specific for the disease, or are too temporally variable to precisely reflect the biological state. This is particularly important in early detection, where candidate markers need to be sufficiently robust to achieve the necessary diagnostic performance in a population screening environment that may involve repeat testing over several years.

In this study, we aimed to characterise the longitudinal stability of circulating microRNA profiles in serum from women with no history of cancer over an extended period. We calculated intraclass correlation coefficients (ICC) from microRNA measurements by small RNA sequencing. We proceed to investigate the impact of normalization, age, BMI, and other sample collection factors on these measurements. Our investigation reveals that the most abundant circulating miRNAs, which are frequently reported as biomarkers for chronic conditions, are not necessarily the most stable and discriminatory over time. We also observed that a known maternally imprinted genomic region was a major contributor to the portion of the serum microRNAome with the greatest longitudinal stability.

## Results

**Figure 1:**
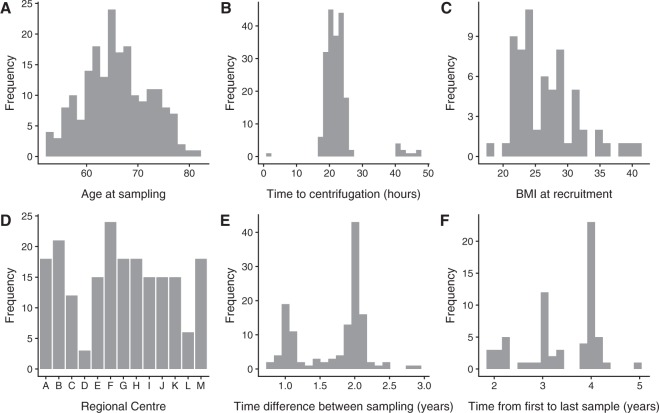
Distributions of study set demographic information. (**A**) age at sampling, (**B**) time to centrifugation, (**C**) BMI at recruitment, (**D**) Regional Centre of collection for our study set, **(E**) time difference between successive samples, and (**F**) time from first to last sample.

Our sample set consisted of 66 women with no cancer from the multi-modal arm of the UKCTOCS cohort for whom 3 serum samples over a ~5-year period was available. Characteristics of the sub-sample are listed in Table 1. The age of individuals at sample collection ranged from 53 to 81 years (Fig. 1A, median 65 years). Time to centrifugation ranged from 1.73 to 47.25 hours after blood collection (Fig. 1B, median 21.65 hours). BMI at recruitment also covers a broad range, from 18.02 to 40.85 (Fig. 1C, median 25.85). These samples were collected at 13 different regional centres (Fig. 1D, between 1 and 8 participants from each). The interval between sampling ranged from 9 months to 3.14 years (Fig. 1E, median 1.96 years), while the interval between the first and third sample ranged from 1.9 to 5.05 years (Fig. 1F).

**Table 1:** Characteristics of the UKCTOCS sub-sample.

	Median	SD	Range
Age at sample collection	65	6.30	53–81
Time to centrifugation (hours)	21.65	5.36	1.73–47.25
BMI at recruitment	25.85	4.84	18.02–40.85
Number in each Regional Centre	5	1.89	1–8

### Abundance and frequency of detection of microRNAs in circulation

**Figure 2:**
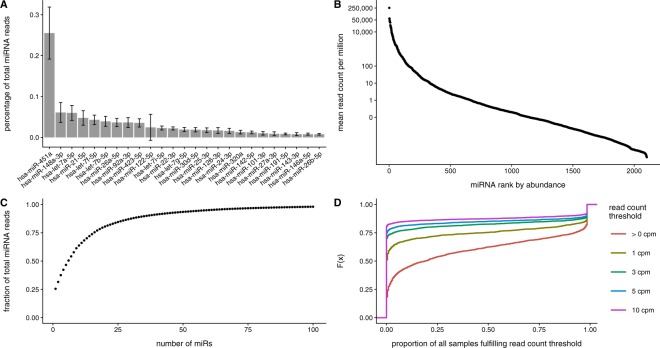
Abundance of miRNAs in circulation. (**A**) The top 20 miRNAs by percentage of total circulating miRNA. (**B**) Mean microRNA counts per million (cpm) in circulation, ordered by magnitude. (**C**) Cumulative fraction of total microRNAs for the top 100 microRNAs by abundance. (**D**) Cumulative distribution of miRNA detection rates at certain cpm thresholds.

The utility of any biomarker candidate depends heavily on our ability to consistently and reliably detect the marker. We examined levels of 2,576 microRNAs annotated in miRBase v20, expressed as read counts per million (cpm). The abundance of each microRNA in circulation was calculated by taking the percentage contribution of each microRNA to total circulating miRNA levels. The top 20 miRNAs by abundance are shown in Fig. 2A below. The most abundant circulating microRNA by a large margin is miR-451a, which on average comprises 25.6% of the serum total. The next most abundant circulating miRNA, miR-146a-3p, only accounts for 6.1% of the total. Individually, the vast majority of miRNAs (1910 out of 2579) are detected at less than 1 cpm (Fig. 2B, Supplementary Table 1). 506 miRNAs are detected at an average of 1–100 cpm, with the remaining 160 detected at over 100 cpm.

Overall, the circulating microRNA profile is dominated by a small number of microRNAs (Fig. 2C). The top 10 microRNAs by abundance account for 60% of the total. The top 36 account for 90%, and the top 100 account for 98% of the total. The vast majority of microRNAs are in the remainder, cumulatively representing only 2% of total circulating microRNA. All microRNA abundances are listed in Supplementary File 1. Red blood cells are notably associated with the most abundant microRNA in serum, miR-451a, as well as the 8^th^ most abundant, miR-92a-3p^21,22^.

As most microRNAs are detected at very low levels, we considered the rate of inclusion in analyses applying a detection filter at thresholds of >0 (i.e. present or absent), 1, 3, 5, and 10 cpm. The cumulative distribution of detection rates at these thresholds are shown in Fig. 2D. The number of microRNAs reaching the detection rates of at least 1 sample, 50%, 75%, 90%, and 100% of samples are listed in Supplementary Table 2. This further underscores the low detection levels of most miRNAs, as over 50% of microRNAs are not detected at a low read count threshold of 1 cpm.

For further analysis, we focused on the 671 miRNAs that are detected (>0 cpm) in at least 90% of samples. This filtering criterion was selected to focus on miRNAs that are very consistently measured in serum, even if they are detected at very low levels.

### Confounding factors affecting microRNA levels

**Figure 3:**
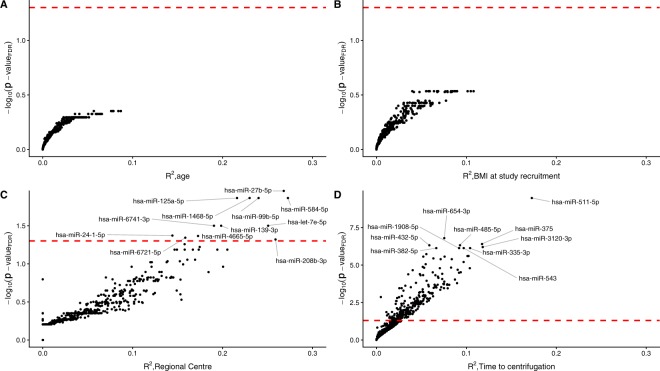
Confounding effects of age, BMI, regional centre, and time to centrifugation. Variance explained (R^2^) against p-values corrected for multiple testing for (**A**) age, (**B**) BMI at study enrollment, (**C**) regional centre of collection, and (**D**) time to centrifugation.

We examined contributions of the confounding factors age, BMI at recruitment, regional centre of collection, and time to centrifugation to microRNA levels. We found no significant associations to age (Fig. 3A) or BMI at recruitment (Fig. 3B). The percentage of variance explained by each factor was low, with age and BMI at recruitment representing under 10% of explained variance for almost all miRs.

We found significant associations to regional centre in 12 microRNAs, explaining between 14.4% and 27.3% of variance (Fig. 3C). These microRNAs (let-7e-5p, miR-125a-5p, miR-139-3p, miR-208b-3p, miR-24-1-5p, miR-4665-5p, miR-584-5p, miR-6721-5p, miR-6741-3p, miR-99b-5p, miR-1468-5p and miR-27b-5p) would presumably be at greater risk of sample collection bias.

Time to centrifugation exerted more significant effects on the microRNA profile (Fig. 3D). More than a third of the microRNAs examined (242 of 684) had significant associations to time to centrifugation, explaining 0.5% to 17.3% of variance in microRNA levels (with a median of 4.1%). We find that effects of time to centrifugation are driven by samples at the extremes of our sample set. While the vast majority of the 198 samples investigated were centrifuged between 17 and 27 hours of collection, there was 1 sample centrifuged 1.73 hours after collection and 10 samples centrifuged 40–48 hours after collection. When we exclude these samples from the analysis, no miRNAs were found to have significant associations to time to centrifugation (Supplementary Fig. 1). When the extremes were excluded, the highest percentage of variance explained for time to centrifugation was 5.5%, lower than that observed for age and BMI at recruitment. 15 miRNAs were associated to regional centre, including the 12 microRNAs associated to regional centre when using the full sample set.

We examined the impact of other normalization on the calculated contribution to variance by examining the effect of using a frequent alternative to total depth normalization, trimmed mean of M (TMM)-normalization (Supplementary Fig. 2). Results were similar compared to total depth-normalized data, with no significant associations to age or BMI at study recruitment. There were 5 miRNAs with a significant regional centre effect in the TMM-normalized data, 1 of which was in common with total depth-normalized data. There were 245 miRNAs associated with time to centrifugation in the TMM-normalized dataset, 222 of which were in common with the total depth-normalized data. Calculated contributions to variance are listed in full in Supplementary File 2.

### Longitudinal stability of microRNA profiles

**Figure 4:**
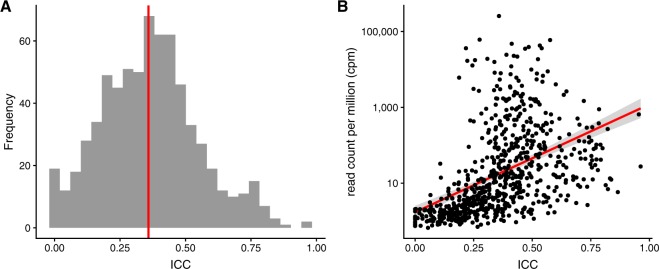
Intra-class correlation coefficients (ICCs) for consistently detected circulating miRNAs. (**A**) Distribution of ICCs for consistently detected miRNAs, with a median ICC of 0.36 shown in red. (**B**) ICCs vs. read counts per million.

We calculated intra-class correlation coefficients (ICCs) to determine the stability of microRNA levels over time (Fig. 4A). The median ICC for the 684 microRNAs examined was 0.361. Two miRNAs, hsa-miR-4326 and hsa-miR-4433b-3p, were found to have excellent reliability (ICC >0.9). 16 miRNAs had good reliability (0.75< ICC <0.9), 123 miRNAs had moderate reliability (0.5< ICC <0.75), and the remaining 543 had poor reliability (ICC <0.5). All ICCs and their 95% CI, as well as the between-person and within-person % coefficient of variance (%CV) for the Top 20 miRNAs by ICC are shown in Table 2 and are listed in full in Supplementary File 3.

**Table 2:** Top 20 miRNAs by Intra-class Correlation Coefficient (ICC).

Rank by ICC	miRNA	read count per million	Within- subject %CV	Between- subject %CV	ICC	95% CI
1	hsa-miR-4326	27.6	21.6	142.4	0.961	(0.941–0.974)
2	hsa-miR-4433b-3p	651.6	48.4	685.4	0.948	(0.922–0.966)
3	hsa-miR-412-5p	5.8	32.6	93.6	0.862	(0.804–0.905)
4	hsa-miR-1255a	12.4	24.5	58.3	0.834	(0.762–0.885)
5	hsa-miR-218-5p	13.4	36.8	92.3	0.829	(0.752–0.880)
6	hsa-miR-7854-3p	7	25.6	57.4	0.818	(0.741–0.873)
7	hsa-miR-370-3p	267.5	32.8	74.9	0.814	(0.732–0.869)
8	hsa-miR-5189-5p	8.9	28.3	61.1	0.804	(0.718–0.861)
9	hsa-miR-493-5p	83.5	34.7	74	0.794	(0.702–0.857)
10	hsa-miR-503-5p	41.5	18	35.5	0.789	(0.700–0.855)
11	hsa-miR-100-5p	890.7	27.4	55.6	0.788	(0.698–0.851)
12	hsa-miR-382-3p	22.1	33	68.1	0.786	(0.692–0.853)
13	hsa-miR-369-3p	30.1	34.5	70.8	0.784	(0.695–0.855)
14	hsa-miR-381-3p	103.6	32	64.7	0.782	(0.690–0.847)
15	hsa-miR-410-3p	42.2	31.5	63.2	0.78	(0.691–0.847)
16	hsa-miR-134-5p	378.2	35.8	67.9	0.759	(0.657–0.837)
17	hsa-miR-494-3p	52.2	33.6	63.3	0.759	(0.669–0.831)
18	hsa-miR-495-3p	119.1	29.3	53.7	0.755	(0.666–0.826)
19	hsa-miR-136-3p	32.1	38.8	71.3	0.746	(0.639–0.827)
20	hsa-miR-127-3p	79.7	36	65.5	0.746	(0.640–0.826)

We found no strong relationship between miRNA abundance and ICCs, as the log-linear regression of mean cpm against ICCs for each miRNA examined (Fig. 4B) had an R^2^ of only 0.214. The Pearson Correlation coefficient was 0.460, also indicating a weak correlation.

**Figure 5:**
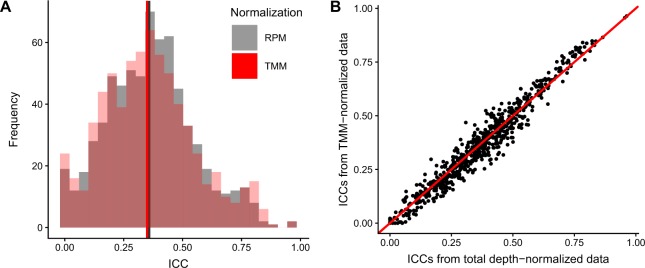
Comparison of ICCs calculated using total depth-normalized data compared to trimmed mean of m (TMM)-normalized data. (**A**) Distributions of ICCs and (**B**) scatter plot of ICCs for each miRNA of total depth-normalized data (as reads per million, RPM) and TMM- normalized data.

We also examined the effect of other normalization on the calculated ICCs. The distribution of ICCs calculated from TMM-normalized data (median 0.350) were very similar to those using total depth-normalized data (median 0.361, Fig. 5A). When comparing ICCs from each normalization for the same miRNA, we found no large deviations in the calculated ICCs between normalization methods (Fig. 5B).

At an average of ~25% of all reads, miR-451a was the most abundant miRNA detected, by a substantial margin. We therefore looked into whether miR-451a levels were exerting an undue impact on the structure of the data after total depth normalization. We found very high correlations between measurements for each miRNA in data after excluding miR-451a and the full total depth-normalized dataset (Supplementary Fig. 3A). This was comparable to correlations between TMM-normalized data and the full total depth-normalized dataset for individual miRNAs, with some lower correlations observed in the TMM-normalized data compared to the miR-451a excluded dataset. The exclusion of miR-451a measurements before total depth-normalization did not substantially alter results of statistical analyses, as ICCs from miR-451a-excluded data were very similar to those calculated using data including all miRNAs before total depth-normalization (Supplementary Fig. 3B).

Given the remarkable stability of a number of the measured miRNAs over the sampling period, we examined whether the most stable microRNAs from two repeat samples could be used to identify the individual from a third, unknown sample. We performed a nearest-neighbour classification using data from 2 samples for each individual in model training, with the remaining third sample for each individual as the test set for prediction. Prediction was carried out over 1500 different permutations of the training set-test set split, and the accuracy of individual ID predictions for the test samples are shown in Table 3. When we used measurements from the 18 miRNAs with ICC ≥0.75, we were able to correctly match miRNA profiles to their corresponding individuals an average of 82.5% of the time. This drops to 73% when we use the 123 miRNAs with ICCs between 0.5 and 0.75, and to 40.8% when we use the 543 miRNAs that have ICCs below 0.5. Interestingly, using all 141 miRNAs with ICC >0.5 produced the highest accuracy, 83.4%, but this was only marginally better than using the 18 miRNAs with the highest ICC. These results indicate that just a small subset of circulating miRNAs are required to stably discriminate between individual phenotypes over extended periods of time.

**Table 3:** Predictive accuracy of nearest-neighbour classification using subsets of detected miRNAs with different ICCs.

Range of ICCs included	Number of miRNAs included	Accuracy of prediction of individual ID (percent)	
		mean	S.D.
ICC ≥0.75	18	82.5	2.6
0.50 ≤ICC ≤0.75	123	73.0	3.4
ICC <0.50	543	40.8	4.1
ICC ≥0.50	141	83.4	3.3
All detected miRNAs	684	67.1	4.4

### Comparison with longitudinal stability in microarray-based measurements

**Figure 6:**
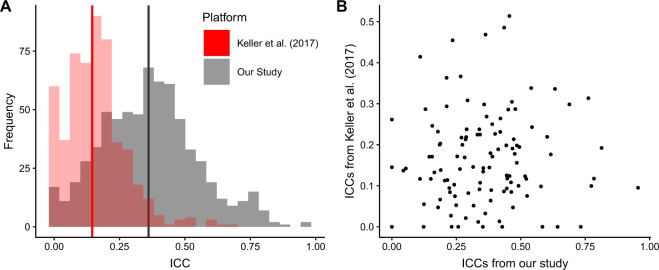
Comparison of ICCs from the longitudinal study carried out by Keller *et al*. (2017) and our own study. (**A**) Distributions of ICCs and (**B**) scatter plot comparing ICCs for 113 miRNAs detected in both studies.

Variability in circulating miRNA signatures was also recently examined in a microarray study with a similar 3-timepoint design^23^. They examined samples from 30 individuals resident in Norway, with ages 30–62 years. Samples were drawn an average of 5 years apart. Using these data (study GSE100768 in the Gene Expression Omnibus), we calculated ICCs for 528 miRNAs that fulfil the inclusion criteria specified by the authors (positive signals in at least 10 samples). Overall, the ICCs we calculated from the Keller *et al*. data were substantially lower than those we observed, ranging from 0 to 0.690, with a median of 0.136 (Fig. 6A). There were 113 miRNAs reported as reliably detected in both studies; comparing the ICCs of these miRNAs we found no clear correlations between the two (Fig. 6B).

### 14q32 is a major source of longitudinally stable circulating microRNAs

**Figure 7:**
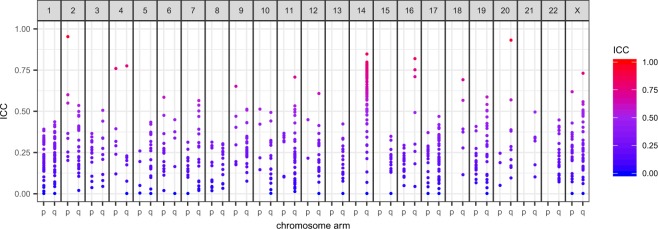
A cluster of miRNAs with ICCs above 0.5 can be found on the long arm of chromosome 14.

An analysis of the ICC values by genome distribution indicated that the long arm of chromosome 14 was enriched in miRNAs associated with high ICC values (Fig. 7). Of the twenty miRNAs with the highest observed ICC in our cohort highlighted in Table 2, 12 originate from the 14q32.2-14q32.31 miRNA cluster *[C14MC, Mirg*^24,25^]. We identified 55 miRNAs we can measure from serum in the DLK1-DIO3 region (chr14: 100726827–101563452), distributed primarily in two neighbouring segments spanning ~40 kb that are part of a maternally-imprinted region of the genome. 47 of these 55 miRNAs possessed an ICC >0.5 in our study, 1/3 of all miRNAs with moderate to high ICC. The median ICC for the cluster was 0.672, significantly higher than that for the whole serum miRNAome (p = 3.3 × 10^−22^ after a Mann-Whitney Test), with the highest ICC for the cluster (miR-412-5p) observed to be 0.863.

## Discussion

The search for circulating microRNA biomarkers of disease continues to be a very active research space. This is largely due to the potential afforded by the presence and apparent stability of microRNA in a range of biofluids, the availability of sensitive detection technologies, and the physicochemical properties that keep microRNAs stable over prolonged periods of time. While hundreds of research papers are published yearly on microRNAs that distinguish individuals with a range of different diseases from healthy controls, these are typically carried out on relatively small case-control studies and have yet to be validated and applied in a broader disease testing context. A key unanswered question is whether we can generalize findings from these case-control study designs to the larger, more diverse general population. A greater understanding of the factors affecting microRNA profiles in healthy individuals over time would allow us to better prioritize miRNAs more likely to be robust discriminants in a population context.

In our study, miR-451a levels were substantially higher than any of the other miRNAs, an observation in some^26,27^ but not all^28^ serum or plasma miRNA sequencing studies. miR-451a is a DICER-independent miRNA^29,30^ required for erythropoiesis^31^ and highly abundant in erythrocytes. Accordingly, high levels of miR-451a, the closely associated miR-144^32^ or the miR-451a/miR-23a-3p ratio from qPCR measurements^33^ in serum or plasma have been suggested to be markers for haemolysis. There are no measures comparable to the miR-451a/miR-23a-3p ratio that have been validated for sequencing data. In our dataset, we calculated the log_2_ miR-451a/miR-23a-3p ratio and found that it was not correlated to pre-analytical factors such as time to centrifugation that might indicate a significant influence of haemolysis in our dataset (Supplementary Fig. 4). We also examined the relationship of serum miR-451a levels to time to centrifugation (Supplementary Fig. 5), and also found that serum levels of miR-451a were not correlated to time to centrifugation in our dataset.

The cellular origin of most microRNAs that can be detected in circulation is currently unknown, although several studies examining miRNA expression in various tissues have been carried out^34–36^. An understanding of the tissue specificity of microRNAs is important in assessing the relevance and utility of any candidate miRNA marker of disease. An up-to-date metric that is readily comparable across different miRNAs and tissues and is based on a broad and diverse base of supporting data would be a key resource in effectively assessing findings from circulating miRNA investigations.

The cell origin of circulating miRNAs is also key to understanding the amounts of miRNA secreted by different cells and tissues into circulation. Levels of a large number of circulating miRNAs have been shown to correlate with blood counts^22^, suggesting that serum miRNA content may reflect perturbations in blood cell counts more strongly than disease effects. Measurements of microRNAs by sequencing are typically represented in relation to total miRNA measured (as reads per million microRNA reads). This is a dependent normalization, and any shift in the read counts of one microRNA will adjust the normalized value for all the other remaining miRNAs even if there has been no change in their absolute expression^37^. As such, any large shifts in levels of certain miRNAs caused by changes in blood cell numbers may substantially influence the miRNA sequencing measurements in serum. This would require much more careful examination of the extent by which blood cell populations (and other contributing tissues) influence the circulating miRNA profile. Future work should also review the longitudinal stability of miRNA isoforms (isomiRs), as these may well encode further information about cellular origin, as shown in a recent study of blood cells^38^.

We found no significant associations to serum miRNA levels for BMI at recruitment. While other studies identify links between circulating miRNA and BMI, our study is limited by the fact that we only have records of BMI at recruitment. Using this assumes that the BMI of subjects remain in a small range of BMI over the period of sampling, and can only inform on relationships to an individual’s tendencies around a certain BMI rather than on changes relating to weight loss or weight gain within individuals.

We also identified no significant associations to age at sampling, which accounted for under 10% of explained variance in the miRNAs examined. This contrasts with previous work where age was associated with 7 out of 179 miRNAs measured by RT-PCR in plasma^39^ from a cohort of 372 individuals ranging in age from 22–79 years. This may relate to differences in the age range explored, as our study is limited to ages between 53 and 85. The identification of stronger age-related effects in a cohort including young individuals may relate to developmental miRNA signatures that differentiate between younger and older individuals. However, as most population screening programmes for diseases such as cancer are likely to be carried out in people above 50 years old, the absence of a strong age-related effect indicates that miRNA measurements in this age group may be robust to impacts from the aging process.

A small number of miRNAs are significantly associated to the regional centre of collection. Although all samples were transported by the next day, the transport time from venipuncture to receipt in central laboratory varied as the centres were located across England, Wales and Northern Ireland. Outside of any difference in blood collection methods, these miRNAs may then potentially relate to other exposures (such as dietary or environmental factors) that are distinct to the different geographic areas. Exposure to dietary components such as isoflavones^40^ and vitamin E^41^, and to environmental chemicals like lead and cadmium^42^, arsenic^43^, and Bisphenol A^44^ have been shown to alter miRNA expression in different cell types, but the impact on circulating miRNA profiles has not been explored. Most miRNA biomarker studies have been carried out as small single-center studies with single point measurements, subjecting them to population biases that limit both genetic and exposure diversity associated with the limited subsample. This increases the possibility of selecting candidate markers that are not sufficiently robust when validating in a broader population, as any confounders relating to geographic or community relationships may no longer be controlled for.

We also found a substantial number of microRNAs that are influenced by time to centrifugation, representing a third of our detected miRNAs. This highlights a need to consider sample collection procedures more carefully in the design of serum miRNA biomarker studies, as these can exert a substantial influence on the microRNA profile. In the top 5 miRNAs affected, time to centrifugation accounts for 10–16% of explained variance, which may be enough to mask the disease discriminant capacity of markers at early stages of disease. However, when we limited the analysis to samples centrifuged 17–27 hours after collection (excluding samples at either extreme), no miRNAs were significantly associated with time to centrifugation. This indicates that serum microRNAs may be robust to impacts of deviations in time to centrifugation during sample processing within that 10-hour period between 17–27 hours, but large variations may be observed with a more extensive range of deviations in sample processing time. This highlights the importance of protocol standardization in these studies, as controlling for these variables experimentally makes a substantial impact on the robustness of the data.

We found no large differences in the contribution of the different confounders examined and in the ICCs when data was TMM-normalized. This indicates that the normalization procedure does not introduce large changes in the variance structure that we would have ascribed to biological or experimental factors in this study.

Our analysis of the long-term stability of serum miRNA profiles from small RNA sequencing identified a small number (25) of miRNAs with good to excellent reliability (ICC ≥0.75). Another 119 miRNAs had moderate reliability (0.50 ≤ICC ≤0.75), with the remainder of consistently detected miRNAs having poor reliability (540, ICC <0.50). The high predictive accuracy of models built on the most longitudinally-stable miRNAs indicate that there is a subset of miRNAs that are characteristic of individuals and do not exhibit large fluctuations over time. While the capacity to uniquely identify individuals on the basis of 25 miRNAs will likely be significantly reduced when expanded to populations in the thousands, it is remarkable that we find such high accuracies of identifying individuals on the basis of two earlier measurements that may be several years apart from the test sample. These findings have positive implications for the likelihood of identifying miRNA biomarkers in serum that report on chronic effects and long-term risk of disease. Such miRNAs may open the possibility for personalized longitudinal screening, where deviations from earlier healthy baseline profiles may indicate the presence of disease. Efforts to achieve this will require small RNA profiling of prospectively collected samples stored for many decades but again the signs are positive; our study involves legacy samples up to 16 years old, while others have reported that concurrent DNA sequencing and sRNAseq is viable in archived serum samples over 40 years old^45^.

Our study is limited by the fact that our cohort is composed entirely of women. This precludes the examination of gender-related effects on circulating miRNA levels. We are also unable to examine circulating miRNA changes that occur dynamically with weight change in individuals, as measurements of BMI at sampling are not available. In addition, while our study is currently one of the larger sample sets measuring longitudinal miRNA profiles in healthy individuals, we are still limited by the number of individuals included (n = 66). It is possible that more significant associations to factors like age and regional centre could be identified with a larger cohort, affording greater statistical power and sample diversity. However, the identification of significant regional centre-related effects in our sample indicates a sufficiently strong role for regional centre of collection, despite our limited sample.

We observed a marked difference in the ICC distributions between the Keller *et al*. study and our own data, which may be effects of differences between miRNA analysis platforms (sequencing, microarray, and PCR) that have been extensively demonstrated in other studies^46^. However, we cannot rule out that these differences in ICCs are in part due to the differences in the study populations. The Keller *et al*. study covers a younger age range (31–62 compared to 53–81) and has, on average, a wider gap between sampling, which may contribute towards increasing the within-individual variability in this sample set. However, the inclusion of both males and females in their study, as well as the smaller sample size, might have been expected to contribute towards increased between-individual variability in their sample.

Our data suggest that the *C14MC* 14q32.2–14q32.31 miRNA cluster is a major source of circulating miRNAs that are stable and discriminating between individuals over extended periods of time, opening the door to monitoring the progression of many important normal and patho-physiological states. Germline mutations and epigenetic alterations in the 14q32.2 imprinted region are associated with developmental defects such as Kagami-Ogata syndrome^47^, but we do not currently know their impact on circulating miRNAs. The *C14MC* microRNA cluster is reported to be actively and selectively exported via exosomes, which may contribute to its ready detection in serum^48^. While detected in a number of tissues including epithelial tissues and brain, *C14MC* miRNAs are frequently reported as highly expressed specifically in embryonic & placental tissue. These miRNAs have therefore been presented as microRNAs associated with, and with potential for monitoring, pregnancy^49^. *C14MC* expression is regulated by differentially methylated regions (DMR) that appear to be sites for maternal imprinting; selective DNA hypermethylation at the DMR prevents expression of the paternal allele^24^. Thus, it may be possible to assess the significance of gene imprinting in the action of *in utero* or post-natal exposures that impact upon health in later life by monitoring the serum expression of miRNAs from this locus. Altered methylation of *C14MC* miRNA coding sequences in lymphocytes has been associated with obesity in childhood^50^, but the relationship of this to serum expression of *C14MC* is currently unknown. Serum levels of cluster miRNAs have been associated with progression of steatosis to non-alcoholic steatohepatitis in mice and humans^51^. The *C14MC* cluster has also been associated with the presence and progression of several malignancies^52–55^, supporting the case for further investigation of the utility of C14MC miRNAs in serum for cancer diagnosis and prognostication.

In the current work, it was not possible to determine the major drivers for differential expression of *C14MC* miRNAs in our cohort. Given the association of the cluster with pregnancy, and that all our data are from females, it is possible that pregnancy history has an influence on our specific dataset. Also, no genetic data was available for our cohort to assess the impact of germline variation on the *C14MC* expression profile. The role of imprinting in this genome region suggests that epigenetics and other drivers of phenotypic variation might be more influential. Future work could confirm this through concurrent genome sequencing and epigenetic analysis. In addition, miRNA isoform analysis would inform on any differences relating to nucleotide substitutions within the mature miRNA sequence that are not currently examined in canonical miRNA analyses such this.

In summary, we demonstrated that a subset of miRNAs that are longitudinally stable in serum could be defined from small RNA sequencing data. We found that profiles of these longitudinally-stable miRNAs were able to individually discriminate between healthy women over a period of up to 5 years. These miRNAs were dominated by those originating from the *C14MC* cluster that is known to be subject to maternal imprinting. This assessment was not significantly affected by common confounders such as age, BMI or time to centrifugation nor alternative methods to data normalisation. Our analysis provides important benchmark data supporting the development of miRNA biomarkers for the impact of life-course exposures as well as the diagnosis and prognostication of chronic disease.

## Materials and Methods

### Study set

We examined a subset of serum samples collected during the course of the UK Collaborative Trial of Ovarian Cancer Screening (UKCTOCS), a 13 centre, randomised controlled trial investigating the impact of ovarian cancer screening on disease mortality. Details of the trial design have been outlined in previous publications^56^. UKCTOCS participants were post-menopausal women aged 50–74, with no active malignancy and no previous history of ovarian cancer at recruitment between 2001 and 2005. In all, 202,638 post-menopausal women were randomly assigned (2:1:1 ratio) to routine care (control; n = 101,359), annual screening using serum cancer antigen 125 (CA125) (multimodal screening, n = 50,640) or transvaginal ultrasound (n = 50,639). All participants were linked using their National Health Service number to national cancer and death registry electronic health records as well as hospital episode statistics (for those resident in England). In addition, women were sent two follow-up questionnaires, the most recent in 2014.

All women provided a blood sample at recruitment with women randomised to the multimodal screening group (n = 50,640) continuing to donate serum annually for up to 11 years from randomisation. Sample collection stopped at the end of screening in December 2011^57^. Blood samples were collected in gel tubes (8 mL gel separation serum tubes; Greiner Bio-One 455071, Stonehouse, UK) at the trial centres and transported at room temperature to a central laboratory^58^. Samples were centrifuged at 1500 *g* for 10 min and the separated serum aliquoted into 10 × 500 μL straws (IMV) using a semi-automated MAPI platform (IMV). The straws were stored in liquid nitrogen tanks at the central laboratory which when full were transported to a HTA licensed, ISO-accredited commercial cryofacility (Fisher Bioservices, UK).

The current study uses a subset of 66 UKCTOCS participants with samples collected at three time points over a period of up to 5 years. All individuals included in the current study had no past or present record of any cancer apart from non-melanoma skin neoplasms (ICD-10 C44*) and other benign conditions based on Health and Social Care Information Centre cancer and death registration data obtained on June 17, 2014 (England and Wales), and July 2, 2014 (Northern Ireland) and Hospital Episode Statistics (HES) data 2000–2010.

### Ethical approval

UKCTOCS was approved by the UK North West Multicentre Research Ethics Committee (North West MREC 00/8/34) with site specific approval from the local regional ethics committees and the Caldicott guardians (data controllers) of the primary care trusts. All women gave informed written consent for use of samples and data in ethically approved secondary studies undertaken in collaboration with academia and/or industry. Research was performed in accordance with the relevant guidelines and regulations. Analysis of the subset of samples used for the present study undertaken in collaboration with CRUK has been approved by the South Central-Hampshire B NRES Committee (REC Ref 14/SC/1323).

### RNA isolations and QC

Aliquots of selected UKCTOCS Biobank human serum samples were sent to Asuragen, Inc. (Austin, Texas, USA), for RNA extraction, NGS-based microRNA profiling, and data analysis for biomarker discovery. All serum samples were visually inspected for evidence of hemolysis before sample processing. Total RNA including small RNAs was isolated by Asuragen from 0.55 mL Process Controls and UKCTOCS Biobank human serum samples according to a proprietary procedure developed by Asuragen for downstream small RNA sequencing. The isolation procedure was based on the miRVana™ PARIS™ Kit published protocol for extraction of total RNA enriched for small RNAs (Thermo Fisher Part Number 1556 M Rev C, 01/2011), with modifications for maximal RNA recovery and yield. Eluted RNA samples were precipitated and resuspended in a volume of 7.5 microliters nuclease-free water each. One microliter of each RNA sample was removed for quality control (QC), and the remaining RNA was stored frozen at −80 °C. Relative miRNA recovery was assessed for QC on the RNA input equivalent of (8) microliters of serum per sample, using single-target RT-qPCR analysis for an endogenous miRNA demonstrating abundant expression in a broad range of samples types as determined by Asuragen. Of note, this method of QC has been validated by Asuragen for prediction of RNA performance in miRNA RTqPCR and microarray applications, but evaluation for miRNA NGS applications was prospective at the time of this study. Inputs for RT-qPCR or library prep are expressed as serum equivalents (SE), reflecting the serum equivalent volume (mL) of isolated and precipitated RNA.

### Library preparation and microRNA sequencing

A volume of isolated and precipitated RNA equivalent to 0.44 mL of serum were used for the generation of small RNA libraries. Library preparations were assessed for purity using an Agilent 2100 BioAnalyzer or Calper LabChip GX before and after size selection for small RNAs. Size selection for miRNAs was performed using a Pippin Prep instrument (Sage Science) with 3% agarose gels. Libraries were quantified using KapaQuant qPCR (Kapa Biosystems). Small RNA libraries were then sequenced with 50 bp single-end reads on an Illumina HiSeq2000 instrument.

### Sequence mapping and miRNA annotation

FASTQ files for each small RNA library were stripped of adapter sequence using cutadapt 1.2.1 and aligned to the human reference genome (Ensembl hg19) using Bowtie 0.12.9. miRNA quantification was performed using htseq-count 0.6.1 using mature miRNA annotations in miRBase v20. miRNA expression data was normalized by the total number of reads per million mapped (RPM) to mature miRNA sequences for a given library.

### Statistical analysis

All statistical analyses were carried out in the R statistical environment (R 3.3.1).

The contribution of the confounding factors age, body mass index (BMI) at recruitment, regional centre of collection, and time to centrifugation were evaluated using a linear mixed effects model including the aforementioned factors as fixed effects and individual IDs as a random effect, with microRNA levels on a log_e_ scale. Linear mixed effects models were fit using the *lme4* package. Variance explained (R^2^) by each factor was calculated using the method described by Nakagawa and Schielzeth^59^ using the package *r2glmm*. The statistical significance of fixed effects were assessed by F-tests based on Kenward-Roger’s approximations for denominator degrees of freedoms using the package *lmerTest*, while random effects were assessed by a restricted likelihood ratio test based on 100,000 simulations from the finite sample distribution using the *exactRLRT* function from the package *RLRsim*.

Processed microarray data from the Keller, *et al.*^23^ study were downloaded from the Gene Expression Omnibus (GEO) under reference number GSE100768.

The between- and within-subject CVs were calculated as the square root of the between- and within-subject variance components from a mixed effects model with the timepoint order as a fixed effect and individual IDs as a random effect, with microRNA levels on a log_e_ scale. Intra-class correlation coefficients (ICCs) were calculated by dividing the between-subject variance by the total variance (the sum of between-subject and within-subject variances). We interpreted ICCs according to the guidelines suggested by Koo and Li^60^: ICC >0.9 – excellent reliability, ICC <0.9 and ICC >0.75 – good reliability, ICC <0.75 and ICC >0.5 – moderate reliability, and ICC <0.5 – poor reliability. 95% CIs of ICCs were calculated from a bootstrapped distribution with 1000 iterations.

## Supplementary information

Supplementary Tables and Figures

Supplementary Dataset 1

Supplementary Dataset 2

Supplementary Dataset 3

## Data Availability

Sequence data have been deposited at the European Genome-Phenome Archive (EGA), which is hosted by the EBI and the CRG, under Accession Number EGAS00001003221.
