# Supplementary material for: The 14q32 maternally imprinted locus is a major source of longitudinally stable circulating microRNAs as measured by small RNA sequencing
Source: Sci Rep. 2019 Oct 31;9:15787. doi: 10.1038/s41598-019-51948-6 (PMC6823392; doi:10.1038/s41598-019-51948-6)
Supplement: Supplementary file 1 — Supplementary Tables and Figures [file 41598_2019_51948_MOESM1_ESM.pdf]

SUPPLEMENTARY TABLES AND FIGURES

The 14q32 maternally imprinted locus is a major source of longitudinally stable circulating microRNAs as measured by small RNA sequencing

Gabriel N Valbuena, Sophia Apostolidou, Rhiannon Roberts, Julie Barnes, Wendy Alderton, Lauren Kerr, Ian Jacobs, Usha Menon, and Hector C Keun

Supplementary Table 1. Number of microRNAs measured at different detection ranges

Percentage of total miRNA	Range of read counts (in counts per million)	number of miRNAs
> 10%	> 100,000	1
1 - 10%	10,000 - 100,000	18
0.5 - 1%	5,000 - 10,000	12
0.1 - 0.5%	1,000 - 5,000	37
0.01 - 0.05%	100 - 1,000	92
0.001 - 0.01%	10 - 100	173
0.0001 - 0.001%	1 - 10	333
0.00001 - 0.0001%	0.1 - 1	479
< 0.00001%	0 - 0.1	1431

Supplementary Table 2. Number of microRNAs fulfilling detection criteria at different read count thresholds

Read count (cpm) threshold	At least 1 sample	At least 50% of samples	At least 75% of samples	At least 90% of samples	In all samples
> 0	2106 (81.8%)	978 (38.0%)	800 (31.1%)	684 (26.6%)	467 (18.1%)
1	1260 (48.9%)	637 (24.7%)	546 (21.2%)	466 (18.1%)	359 (13.9%)
3	767 (29.8%)	447 (17.4%)	395 (15.3%)	360 (14.0%)	289 (11.2%)
5	644 (25.0%)	387 (15.0%)	351 (13.6%)	320 (12.4%)	260 (10.1%)
10	490 (19.0%)	325 (12.6%)	290 (11.3%)	260 (10.1%)	212 (8.2%)

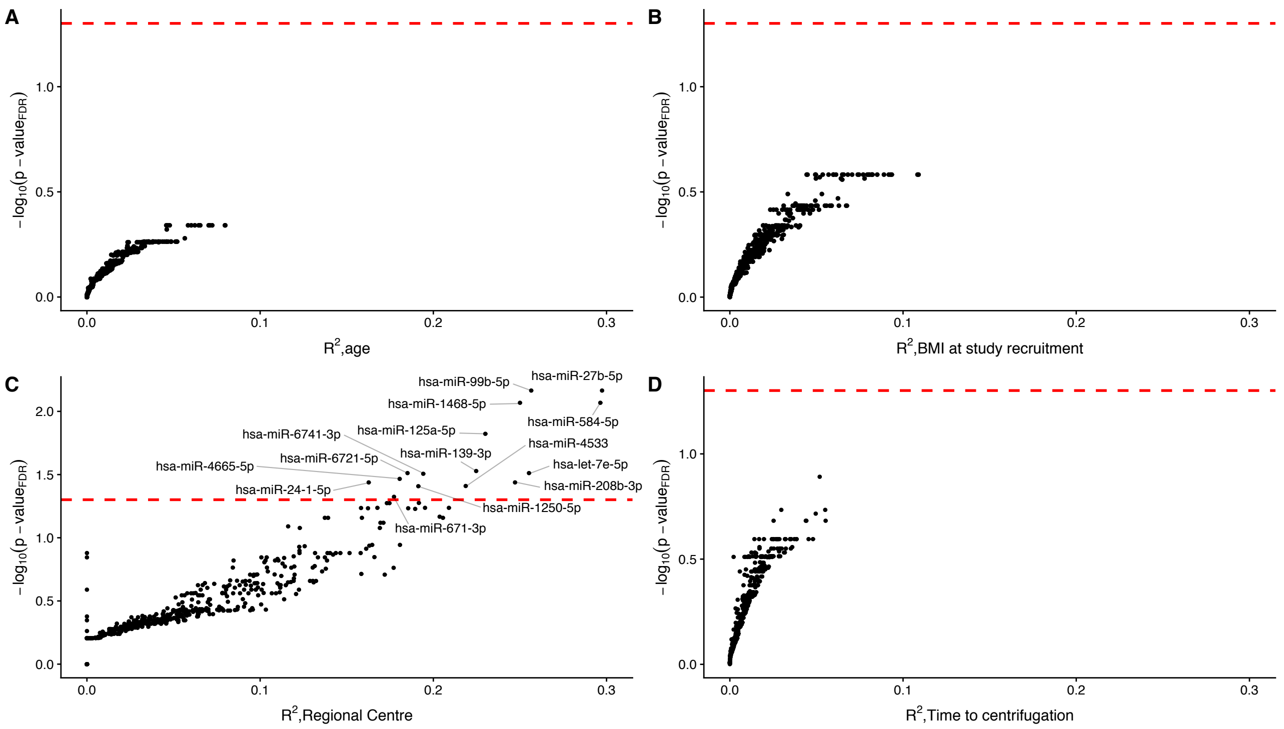

Supplementary Figure 1. Confounding effects of age, BMI, regional centre, and time to centrifugation when excluding centrifugation times under 15 hours and above 40 hours past sample collection. Variance explained (R^2) against p-values corrected for multiple testing for (A) age, (B) BMI at study enrollment, (C) regional centre of collection, and (D) time to centrifugation.

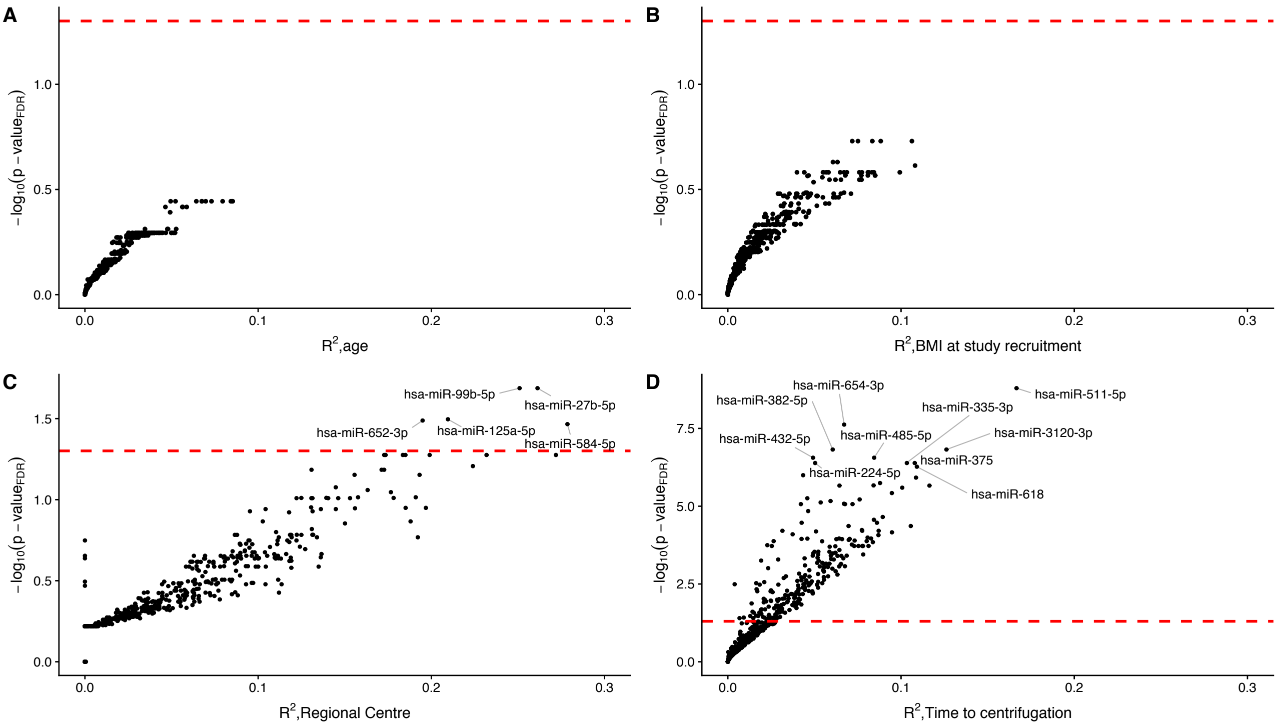

Supplementary Figure 2. Confounding effects of age, BMI, regional centre, and time to centrifugation in TMM-normalized data. Variance explained (R^2) against p-values corrected for multiple testing for (A) age, (B) BMI at study enrollment, (C) regional centre of collection, and (D) time to centrifugation.

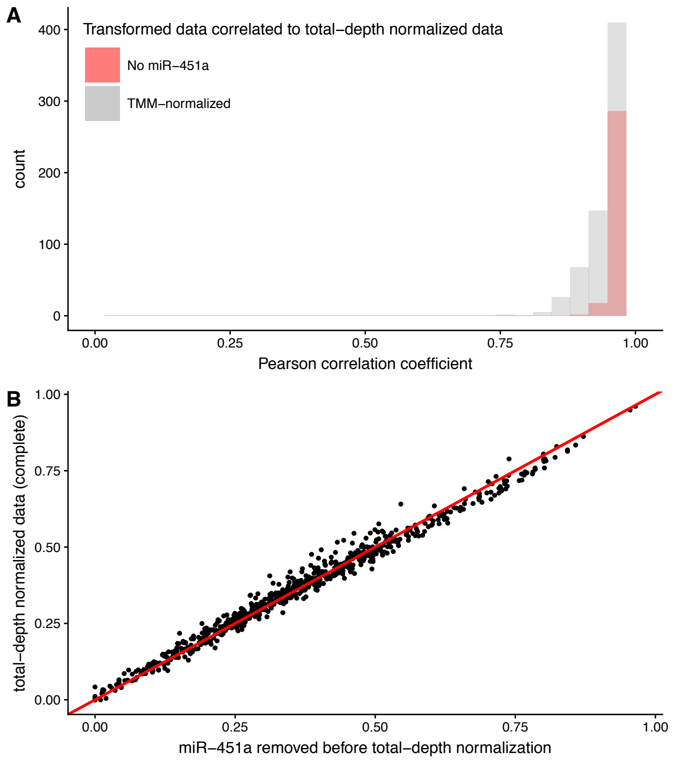

Supplementary Figure 3. Impact of miR-451a measurements on the structure of the miRNA dataset. (A) The distribution of Pearson correlation coefficients for correlations between miRNA data excluding miR-451a before total depth-normalization (in red) or TMM-normalized miRNA data (in grey) and the full total depth-normalized miRNA dataset. When miR-451a is excluded before normalizing to total read depth, the remaining measurements continue to be highly correlated to the full dataset where miR-451a was not excluded before normalization, and it does not exert a greater impact on the data than TMM-normalization. (B) ICCs calculated using the full total-depth normalized dataset compared to when miR-451a is excluded. No substantial deviations in the calculated ICC are observed when miR-451a is excluded.

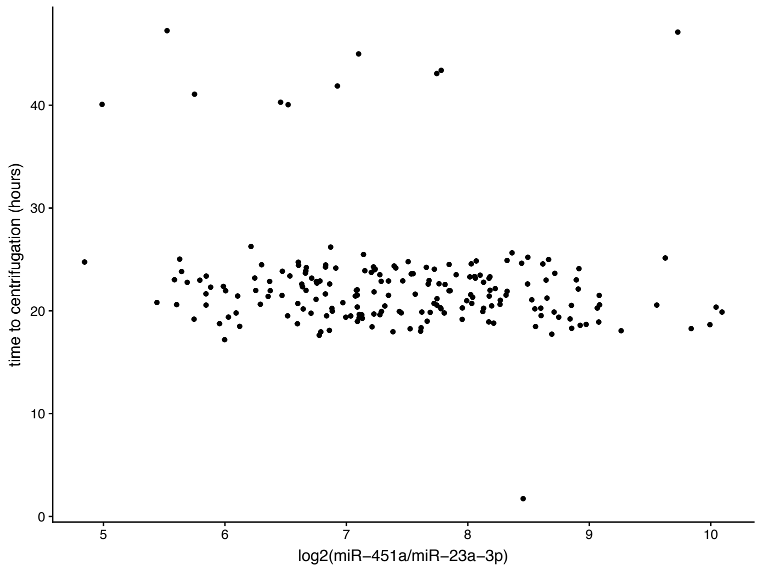

Supplementary Figure 4. No correlation between log2(miR-451a/miR-23a-3p) ratio and time to centrifugation can be observed from our serum miRNA sequencing data.

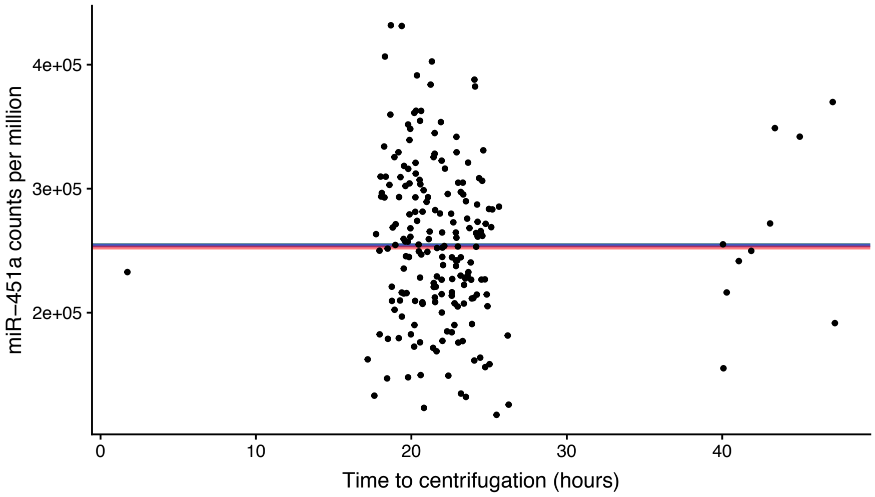

Supplementary Figure 5. No correlation between serum miR-451a levels and time to centrifugation can be observed from our serum miRNA sequencing data. Figure shows serum miR-451a levels (counts per million) against time to centrifugation (hours) for the samples included in the study. We observe no substantial difference between median serum miR-451a levels for all samples (254,204.1 cpm, shown by the blue line), for samples centrifuged 17-27 hours after collection (254,623.6, shown by the green line, and overlapped by the blue line), and for samples centrifuged > 27 hours after collection (252,514.1, shown by the red line), and no clear shift in the distribution of serum miR-451a levels of samples centrifuged > 27 hours after collection relative to those centrifuged 17-27 hours after collection.
